# *Gardenia Jasminoides* Ameliorates Antibiotic-Associated Aggravation of DNCB-Induced Atopic Dermatitis by Restoring the Intestinal Microbiome Profile

**DOI:** 10.3390/nu13041349

**Published:** 2021-04-18

**Authors:** Hyo In Kim, Se Hyang Hong, Seo Yeon Lee, Jin Mo Ku, Min Jeong Kim, Seong-Gyu Ko

**Affiliations:** 1 Department of Surgery, Beth Israel Deaconess Medical Center/Harvard Medical School, 330 Brookline Ave, Boston, MA 02215, USA; hyoin0428@gmail.com; 2 Clinical Medicine Division, Korea Institute of Oriental Medicine, 1672 Yuseongdae-ro, Yuseong-gu, Daejeon 34054, Korea; sehyanghong@gmail.com; 3 Department of Science in Korean Medicine, Graduate School, Kyung Hee University, 26 Kyungheedae-ro, Dongdaemun-gu, Seoul 02447, Korea; dltjdus0225@naver.com (S.Y.L.); jung8328@hanmail.net (M.J.K.); 4 Department of Preventive Medicine, College of Korean Medicine, Kyung Hee University, 26 Kyungheedae-ro, Dongdaemun-gu, Seoul 02447, Korea; saory_ykm@naver.com; 5 Korean Medicine-Based Drug Repositioning Cancer Research Center, College of Korean Medicine, Kyung Hee University, 26 Kyungheedae-ro, Dongdaemun-gu, Seoul 02447, Korea

**Keywords:** intestinal microbiome, atopic dermatitis, *Gardenia jasminoides*, interleukin-17, Firmicutes, Bacteroidetes

## Abstract

The intestinal microbiome is considered one of the key regulators of health. Accordingly, the severity of atopic dermatitis (AD) is mediated by the skin and intestinal microbiome environment. In this study, while evaluating the aggravation in AD symptoms by the antibiotics cocktail (ABX)-induced depletion of the intestinal microbiome, we sought to verify the effect of *Gardenia jasminoides* (GJ), a medicinal herb used for inflammatory diseases, on AD regarding its role on the intestinal microbiome. To verify the aggravation in AD symptoms induced by the depletion of the intestinal microbiome, we established a novel mouse model by administrating an ABX to create a microbiome-free environment in the intestine, and then applied 2,4-dinitrochlorobenzene (DNCB) to induce an AD-like skin inflammatory response. While ABX treatment aggravated AD-like symptoms, the 2-week administration of GJ improved these pathological changes. DNCB application upregulated immune cell count and serum cytokine expression, which were alleviated by GJ. Moreover, pathological alterations by antibiotics and DNCB, including histological damage of the intestine and the intestinal expression of IL-17, were recovered in GJ-treated mice. The beneficial effect of GJ was due to the restoration of the intestinal microbiome composition. Overall, we suggest GJ as a potential therapeutic agent for AD due to its regulation of the intestinal microbiome.

## 1. Introduction

The intestinal microbiome is closely related to the balance between health and disease, including the onset of immune activation and the development of inflammation. This is because the composition of the host microbial community is involved in homeostasis [1]. The microbiome contributes to host health by colonizing the mucosal entry sites of pathogens, and thus regulates effective innate anti-viral immunity through the induction of the interferon (IFN)-λ and IL-18 or IL-22 pathways [2]. Communication among local microorganisms stimulates epithelial and immune cells to secrete IL-22, strengthening the epithelial barrier through interactions with mast cells, eosinophils, basophils, and macrophages, which then initiate antibacterial programs and activate neutrophils through IL-17 secretion [3]. By such mechanisms, the gut microbiota plays an important role in inflammatory and autoimmune diseases. Research on microbial populations and the functional properties on mucosal immunity and distal inflammation has gained interest in the treatment of inflammatory and autoimmune diseases [4].

Since whole-body inflammation is closely related to the pathology of AD, it is logical to assume that the participation of the intestinal microbiome may be important during the aggravation of AD. In a previous study, the Chao1 index revealed the differences in the composition of stool microbial groups between normal control and atopic dermatitis (AD) individuals [5]. In addition to its effect on mast cells, eosinophils, basophils, and macrophages, it is also known that the intestinal mucosal immune system contributes to immune antigenicity by regulating the ratio of regulatory T (Treg) cells to effector T cells [6]. In addition, it is possible to obtain an anti-inflammatory effect from the free fatty acids produced by intestinal microorganisms [7]. As a result, it was suggested that dysbiosis of the intestinal microflora is related to AD development.

AD, a chronic skin inflammatory disease, occurs as a result of a Th2-mediated immune response to constant allergen exposure [8]. It is generally characterized by the overexpression of Th2-type cytokines and is associated with increased IgE and eosinophils [9]. *Gardenia jasminoides* (GJ) has been reported to lower inflammation-related cytokine levels, including IL-6, IL-12, TNF-α, and IFN-γ [10]. In addition, studies have shown that its plasma metabolites affect the composition of the intestinal microbiota [11,12]. Hence, this study aimed to verify the role of the intestinal microbiota in AD through a newly established Antibiotics Cocktail (ABX) model accompanied by DNCB treatment. This experimental model in mice displayed disintegrate intestinal microbiota with classical AD symptoms. Then, the effect of GJ in the same model was evaluated, focusing on how GJ restored the balance of the intestinal microbiota and consequently affected the symptoms of AD.

## 2. Materials and Methods

### 2.1. Preparation of Gardenia jasminoides (GJ)

GJ powder (provided by Hanpoong Pharmaceutical Co., Jeonju, Korea) was dissolved at 200 mg/kg in drinking water for the in vivo study.

### 2.2. Chromatography Analysis of GJ

The high-resolution analysis of GJ was performed with a liquid chromatography-mass spectrometry (LC-MS) system consisting of a Thermo Scientific Accela-HPLC System (composed of Accela 80 Hz PDA Detector (200 to 800 nm), Accela Autosampler, and Model 600 Pump) (Thermo Fisher Scientific, Waltham, MA, USA) and a Thermo Scientific LTQ Velos Mass Spectrometer System (Thermo Fisher Scientific, Waltham, MA, USA). The specific conditions of the analysis are noted in Table 1.

### 2.3. Animal Experiments

**Figure 1:**
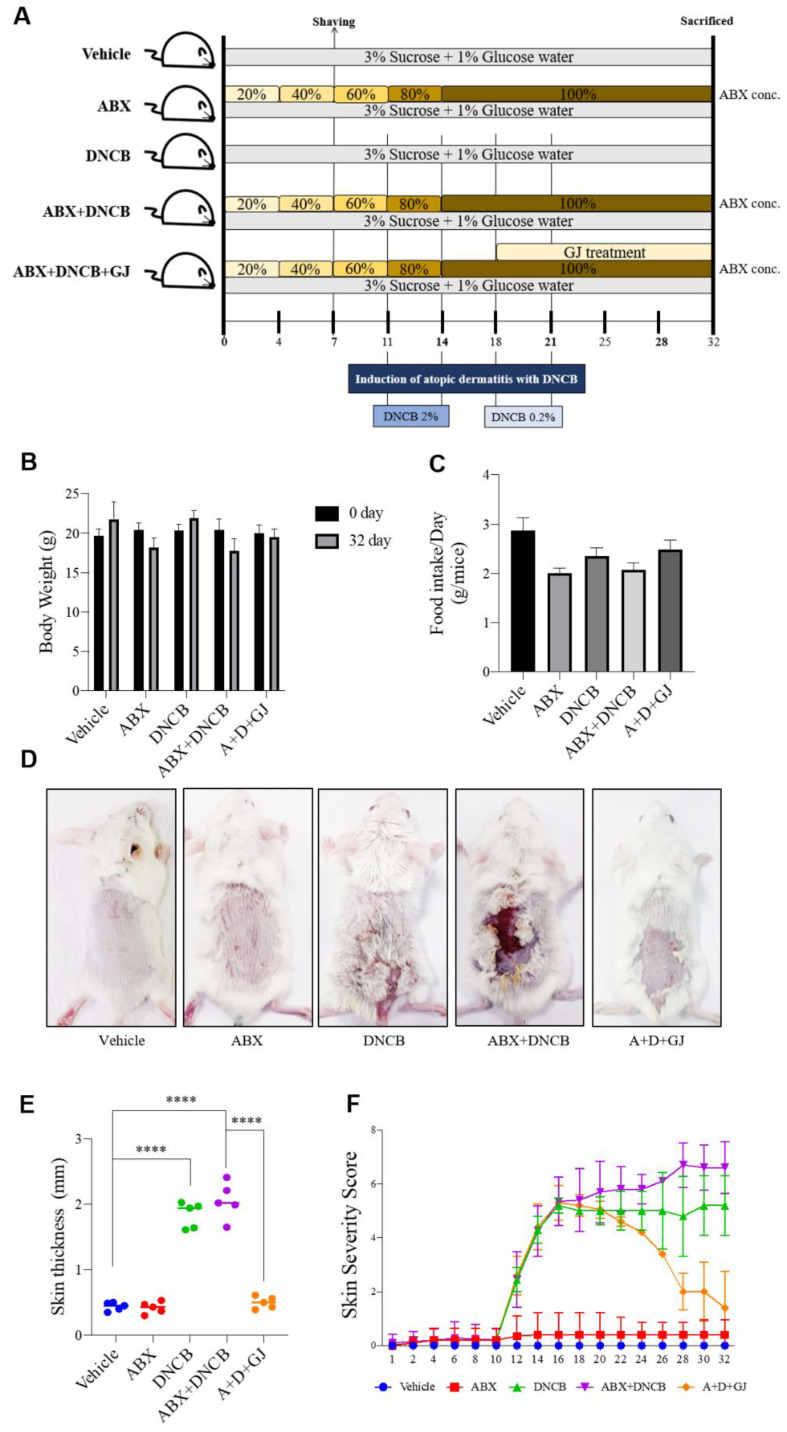
Effect of GJ on AD-related symptoms in ABX-induced AD mice. (**A**) Experimental scheme of animal study. Time-dependent changes in (**B**) body weight and (**C**) food intake per day (g/mice) were measured. Vehicle: normal control group; ABX: antibiotic cocktail-administered group. (**D**) Visual observation of dorsal skin was made and (**E**) the dorsal thickness was measured using a Vernier caliper. (**F**) The skin severity score was visually evaluated between 1 and 10 points. Vehicle: normal control group; ABX: antibiotic cocktail-administered group; DNCB: DNCB-applied group; ABX+DNCB: antibiotic cocktail-administered and DNCB-applied group; A+D+GJ: antibiotic cocktail-administered and DNCB-applied group fed with GJ. **** p < 0.001.

Four-week-old male BALB/c mice (20 ± 2 g) were purchased from Nara Biotech (Seoul, Korea). Mice were housed in a pathogen-free environment and provided ad libitum access to food and water. After 1 week of acclimatization, mice were randomly distributed into five groups (*n* = 5 per group). All methods were performed according to the mouse-related guidelines approved by the Kyung Hee University Animal Care Center (approval number: KHUASP (SE)-18–144). Body weight and food intake were measured every other day. Cages were changed once a week, and fecal samples were collected during the cage changes. The induction of AD was performed by applying 2,4-dinitrochlorobenzene (DNCB) to the shaved dorsal region of mice for a total of 4 sensitizations. The vehicle group and DNCB group were fed with normal chow diet and drinking water containing 3% sucrose and 1% glucose. The ABX group, ABX+DNCB group, and ABX+DNCB+GJ group were administrated a normal chow diet and drinking water containing 3% sucrose and 1% glucose and ABX, of which the concentration was gradually increased from 20% to 100%. The ABX was composed of ampicillin (A9393) 1 g/L, metronidazole (M3761) 1 g/L, vancomycin (94747) 0.5 g/L, and neomycin sulfate (Calbiochem 1405–10-3) 1 g/L based on a previous study [13], all of which were purchased from Sigma-Aldrich (St. Louis, MO, USA). The concentration of antibiotics was gradually increased (20–100%) over time. GJ was orally administrated at 200 mg/kg every other day for two weeks. The experimental scheme is displayed in Figure 1A. CO_2_ inhalation was used to euthanize mice at the end of all experiments, after which cardiac blood and tissues were collected.

### 2.4. Enzyme-Linked Immune Sorbent Assay (ELISA)

Serum IL-4, IL-6, IL-12, IL-13, IL-17, IL-22, TNF-α, and IgE were measured using ELISA kits from BD Biosciences (San Jose, CA, USA) according to the manufacturer’s instructions.

### 2.5. Reverse Transcription Polymerase Chain Reaction (RT-PCR) Analysis

The easy-BLUE™ Total RNA Extraction Kit (iNtRON Biotech, Korea) was used for the extraction of total RNA. An RNA concentration of 1 µg was used for cDNA preparation with a cDNA synthesis kit (Takara Bio Inc., Kusatsu, Japan), and 1 µg of cDNA was used for the RT-PCR assay with specific primers. Table 2 shows the primers used in this study. Samples were loaded in a 1% agarose gel and visual confirmation was obtained under a UV lamp.

### 2.6. Protein Extraction and Immunoblotting Assay

Harvested tissues were lysed with RIPA buffer (BIOSESANG, Seoul, Korea) to isolate proteins. Isolated proteins or fractions were separated by sodium dodecyl sulfate-polyacrylamide gel electrophoresis (SDS-PAGE) and transferred onto nitrocellulose membranes. Then, the membranes were incubated with the following primary antibodies (1:1000) at 4 °C overnight: glyceraldehyde-3-phosphate dehydrogenase (GAPDH) (#5174; Cell Signaling Technology, Danvers, MA, USA), IL-17RA (bs-2606R; Bioss, Woburn, MA, USA), and IL-17RC (LS-C294856; LifeSpan Biosciences, Seattle, WA, USA). The next day, the membranes were incubated with secondary antibodies diluted at 1:5000 for 1 h at room temperature and immunoblot signals were developed using the Pierce™ ECL Western Blotting Substrate (Waltham, MA, USA).

### 2.7. Hematoxylin and Eosin (H&E) and Toluidine Blue Staining

Nuclei and cytoplasm were stained with H&E solution for histological examination and toluidine blue to identify mast cells. After staining, representative pictures of skin and intestine tissue were obtained with an optical microscope (Olympus). The epidermal thickness was measured using ImageJ software (National Institutes of Health, Bethesda, MD, USA). Intestine injury score was calculated based on the histological scoring system (Table 3) as reported previously [14].

### 2.8. Immunohistochemistry (IHC) Staining

Intestine and skin tissues were fixed in 4% formaldehyde and firmly embedded in paraffin for sectioning at 6 μm. Then, slides were incubated with antibodies for thymic stromal lymphopoietin (TSLP) and Ki-67 (Abcam, Cambridge, UK) at 4 °C. The detection of IHC signals was developed using the Vectastain Elite ABC Kit (Vector Lab, Burling Games, CA, USA). Images were obtained with a DM2500 microscope (Leica, Wetzlsar, Germany).

### 2.9. Whole Blood Immune Cell Count

After collecting a whole blood sample by cardiac puncture, the blood was placed in a Vacutainer tube containing EDTA (BD Biosciences, San Jose, CA, USA). Then, a blood analyzer (Hemavet 950, Drew Scientific, Germany) was used to analyze white blood cells (WBCs), particularly neutrophil and monocyte numbers in whole blood.

### 2.10. Fecal Occult Blood Test

Fecal samples were collected, and a thin smear was applied inside a guaiac paper box. Two drops of Hemoccult Sensa^®^ Developer were applied directly over each smear and the result was obtained in 1 min. A blue color indicates that the fecal sample contains the hemoglobin-derived catalyst. Scoring was evaluated as per the manufacturer’s manual.

### 2.11. Intestinal Microbiota Analysis

#### 2.11.1. Metagenome (16s rRNA)

A 16S rRNA sequencing library was constructed according to the 16S metagenomics sequencing library preparation protocol (Illumina, San Diego, CA, USA) targeting the V3 and V4 hypervariable regions of the 16S rRNA gene. KAPA HiFi HotStart ReadyMix (KAPA Biosystems, Wilmington, MA, USA) and the Agencourt AMPure XP system (Beckman Coulter Genomics, Brea, CA, USA) were used for PCR and purification of the PCR product, respectively. The initial PCR was performed with 12 ng of template DNA using region-specific primers (Forward: 5′-TCG TCG GCA GCG TCA GAT GTG TAT AAG AGA CAG TCG TCG GCA GCG TCA GAT GTG TAT AAG AGA CAG CCT ACG GGN GGC WGCAG-3′; Reverse: 5′-GTC TCG TGG GCT CGG AGA TGT GTA TAA GAG ACA GGT CTC GTG GGC TCG GAG ATG TGT ATA AGA GAC AGG ACT ACH VGG GTA TCT AATCC-3′). After purification of the PCR products, a second PCR was performed using primers from the Nextera XT Index Kit (Illumina), which were then visualized by gel electrophoresis. Quantification was done with a Qubit dsDNA HS Assay Kit (Thermo Scientific) on a Qubit 3.0 fluorometer. Pooled samples were run on an Agilent 2100 bioanalyzer (Agilent) for quality analysis prior to sequencing. Libraries were quantified by qPCR using CFX96 Real Time System (Biorad). After normalization, sequencing of the prepared library was conducted on the Miseq system (Illumina) with 300 bp paired-end reads.

#### 2.11.2. Pre-Processing of Sequencing Data

Sequencing reads were sorted by bcl2fastq2 software with unique barcodes. The barcode, linker, and primer sequences were then removed by CutAdapt v1.11, and the removed reads were merged with paired-end reads using FLASH v1.2.11. Merged reads that fit the following criteria were filtered out: reads containing two or more ambiguous nucleotides, reads with a low-quality score (average score < 20), or reads shorter than 300 bp. Potential chimeric sequences were detected with ChimeraSlayer r20110519.

#### 2.11.3. Taxonomy Profiling

The pre-processed reads from each sample were used to calculate the number of operational taxonomic units (OTUs). The OTU number was determined by clustering the sequences from each sample using a 97% sequence identity cut-off with QIIME software (v.1.8.0). Taxonomic abundance was counted with RDP Classifier v2.11 using a confidence threshold of 0.8 from the pre-processed reads for each sample, and NCBI Blast v2.2.28 following clustering by CD-HIT v4.6 using a 99% sequence identity with 80% read coverage cut-off. The microbial composition was normalized by the ratio of taxonomy abundance count and the number of pre-processed reads.

#### 2.11.4. Statistical Analysis

The OTUs were analyzed using the Shannon index to measure the alpha diversity of each sample. The difference in organism composition was measured according to the Bray–Curtis distance to measure beta diversity. Principal component analysis was performed using the beta diversities.

### 2.12. Statistical Analysis

Results are expressed as the mean ± standard error (S.E.) of three or more experiments. Statistical analyses were performed using GraphPad Prism 8 (GraphPad Software, Inc., La Jolla, CA, USA) by one-way ANOVA followed by Tukey’s post-hoc test to determine statistical differences (*p* < 0.05) between groups.

## 3. Results

### 3.1. Gardenia jasminoides Extract Improves AD Symptoms in the Dorsal Skin of DNCB-Applied Microbiome-Deficient Mice

**Figure 2:**
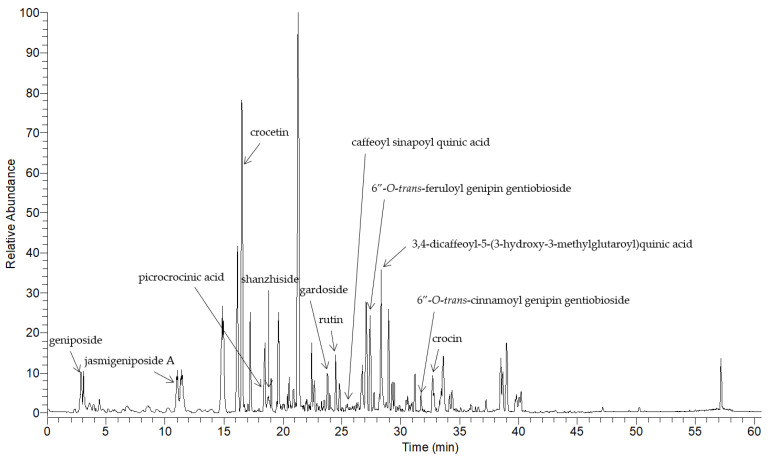
LC-MS-MS analysis of GJ.

To validate the compounds composing GJ, we performed an LC-MS-MS analysis. The chromatography analysis allowed us to identify components including geniposide, jasmigeniposide A, crocetin, picrocrocinic acid, shanzhiside, gardoside, rutin, caffeoyl sinapoyl quinic acid, 6”-O-*trans*-feruloyl genipin gentiobioside, 3,4-dicaffeoyl-5-(3-hydroxy-3-methylglutaroyl) quinic acid, 6”-O-*trans*-cinnamoyl genipin gentiobioside, and crocin (Figure 2 and Table 4).

### 3.2. Gardenia jasminoides Extract Improves AD Symptoms in the Dorsal Skin of DNCB-Applied Microbiome-Deficient Mice

To verify the underlying role of the gut microbiome in AD, an AD mouse model with a depleted intestinal microbiome was produced. ABX was administered to BALB/c mice by gradually increasing the concentration for a total of 32 days. DNCB was applied on the dorsal skin of mice four times for sensitization. To assess its beneficial effect, GJ was orally administrated every other day for 2 weeks. The ABX consisted of ampicillin 1 g, metronidazole 1 g, vancomycin 0.5 g, and neomycin sulfate 1 g in 1 L of drinking water. The concentration of the ABX was gradually increased by 20% every 3–4 days in DW containing 3% sucrose and 1% glucose (Figure 1A).

ABX administration induced weight loss in mice (Figure 1B), probably due to decreased food intake (Figure 1C). While control mice showed a gradual increase in food intake, ABX mice showed decreased food consumption (less than half compared to control mice at the end point of 32 days). However, GJ treatment slightly recovered the body weight lost at the end of the experiment. The clinical symptoms of AD were also evaluated (Figure 1D–F). The depletion of gut microbiota by ABX did not induce any differences in the skin phenotype. However, while DNCB application resulted in AD-like symptoms such as visual condition, thickness, and increases in the severity score of skin, ABX treatment further increased the skin severity score but not the thickness of skin. GJ treatment significantly reduced these symptoms close to the basal level of the control mice.

### 3.3. Gardenia jasminoides Extract Regulates Immune Cell-Related Hematological Parameters and Cytokine Expression in the Serum of DNCB-Applied ABX-Induced Mice

**Figure 3:**
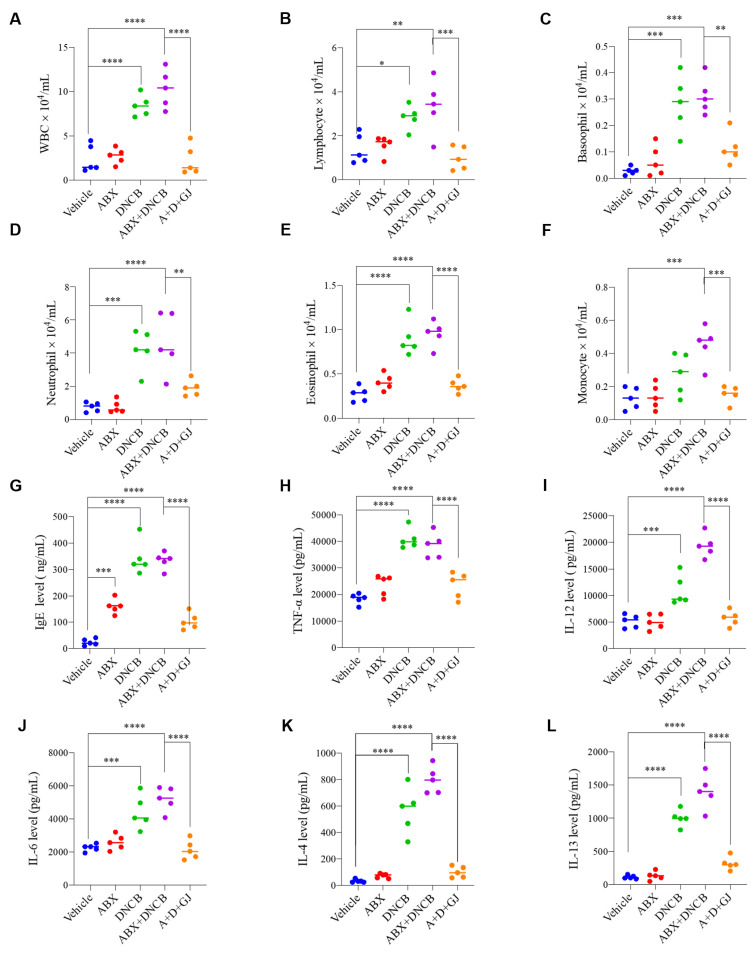
Effect of GJ on blood cell composition and serum cytokine expression in ABX-induced AD mice. The number of (**A**) total WBCs, (**B**) lymphocytes, (**C**) basophils, (**D**) neutrophils, (**E**) eosinophils and (**F**) monocytes was evaluated by a HEMAVET 950 blood analysis system in blood samples from mice. Serum expression level of (**G**) IgE, (**H**) TNF-α, (**I**) IL-12, (**J**) IL-6, (**K**) IL-4 and (**L**) IL-13 were measured by ELISA. Vehicle: normal control group; ABX: antibiotic cocktail-administered group; DNCB: DNCB-applied group; ABX+DNCB: antibiotic cocktail-administered and DNCB-applied group; A+D+GJ: antibiotic cocktail-administered and DNCB-applied group fed with GJ. * p < 0.05, ** p < 0.01, *** p < 0.005 and **** p < 0.001.

An increased number of immune cells in the blood is a classical symptom of AD patients [15,16]. Therefore, after the induction of AD with DNCB in BALB/c mice, the number of immune cells in blood was measured using a HEMAVET 950 hematology analyzer. As shown in Figure 3A–F, depletion of the gut microbiome did not seem to affect immune cell number or composition in blood. However, when AD was induced by DNCB application, the numbers of total WBCs, lymphocytes, basophils, neutrophils, eosinophils, and monocytes were all increased. The number of these immune cells did not significantly differ between mice treated with or without ABX. GJ treatment, on the other hand, significantly reduced the total number of WBCs and WBC subtypes, including granulocytes (neutrophils, basophils, eosinophils), monocytes, and lymphocytes.

The imbalance of cytokines released from T helper (Th) 1 cells and Th2 cells is involved in immunoglobulin E (IgE)-mediated hypersensitivity in the skin; thus, the cytokine balance is considered important in the development of atopic dermatitis. Serum from BALB/c mice with DNCB-driven AD shows increased agglutination of IgE [17]. Tumor necrosis factor (TNF)-α and interleukin (IL)-6 are closely related to the aggravation of AD [18], and IL-12 induces the differentiation of Th1 cells to worsen AD symptoms [19]. The results in this study were also consistent with these previous reports. The key antibody, IgE, along with Th1-type cytokine, TNF-α, and Th2-type cytokines, IL-4, IL-6, IL-12 and IL-13, were measured in mouse serum. The levels of IgE, TNF α, IL-12, IL-6, IL-4 and IL-13 were significantly increased in DNCB-treated mice. Interestingly, IgE was also increased in ABX-fed mice without DNCB application. In GJ-treated mice, the cytokine levels were stabilized and close to the levels of the control groups (Figure 3G–L).

### 3.4. Gardenia jasminoides Extract Reduces Epidermal Thickness, Mast Cell Infiltration and the Expression of Inflammation-Related Markers in the Dorsal Skin of DNCB-Applied ABX-Induced Mice

**Figure 4:**
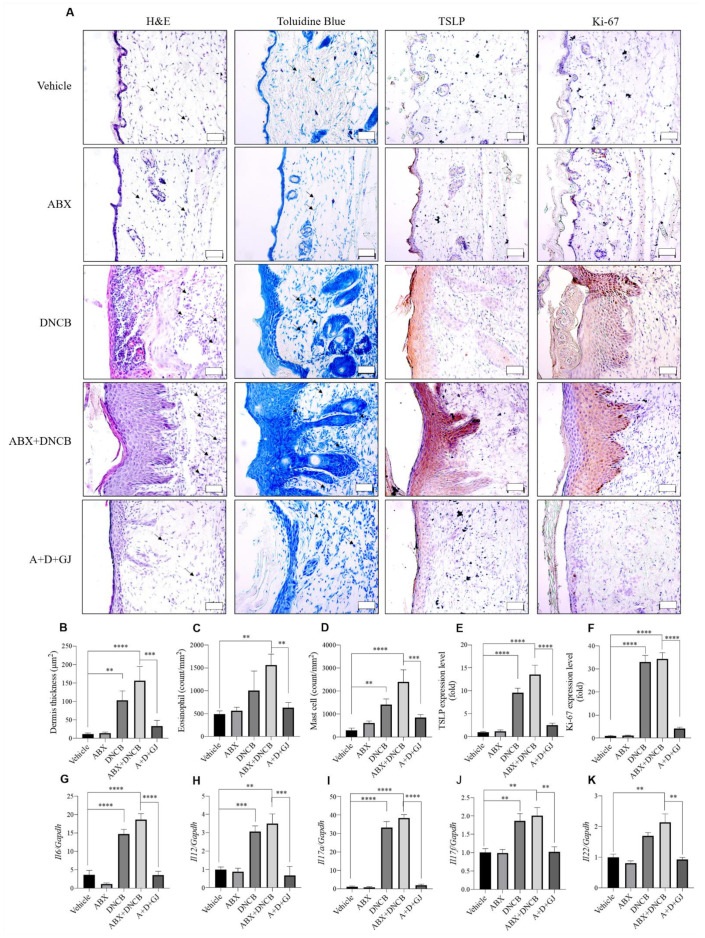
Effect of GJ on histological and immunological factors in the dorsal skin of ABX-induced AD mice. (**A**) Dorsal skins were sliced by paraffin section and stained with H&E to evaluate histological changes and infiltration of eosinophils (indicated by black arrows) (1st column), toluidine blue to evaluate mast cell infiltration (indicated by black arrows) (2nd column), or antibodies for TSLP (3rd column) and Ki-67 (4th column) (magnification ×400). (**B**) Dermis thickness, (**C**) eosinophil count, (**D**) mast cell count, (**E**) relative TSLP expression and (**F**) relative Ki-67 expression were measured. The mRNA levels of (**G**) IL-6, (**H**) IL-12, (I) IL-17A, (**J**) IL-17F and (**K**) IL-22 in skin tissue were measured by RT-PCR. GAPDH was used as an internal control. Vehicle: normal control group; ABX: antibiotic cocktail-administered group; DNCB, DNCB-applied group; ABX+DNCB, antibiotic cocktail-administered and DNCB-applied group; A+D+GJ, antibiotic cocktail-administered and DNCB-applied group fed with GJ. ** p < 0.01, *** p < 0.005 and **** p < 0.001.

As shown in Figure 1, repeated sensitization with DNCB causes AD-related symptoms such as erythema, calibration, dryness, and thickness of the skin. Another important symptom during the development of AD is epidermal proliferation, which is induced by chronic inflammation. The epidermal thickness of dorsal skin was evaluated by H&E staining. As shown in Figure 4A,C, ABX mice did not show any differences from control mice, whereas DNCB application notably increased the thickness of the epidermis in both microbiome-present and microbiome-deficient mice. However, GJ treatment was able to reverse this thickness, although the mice were also sensitized with DNCB. Toluidine blue staining showed increased mast cell numbers in DNCB-induced mice and DNCB-induced ABX mice compared to non-treated mice, indicating mast cell infiltration. However, ABX mice fed with GJ showed a significant reduction in mast cell numbers, similar to those of the control group (Figure 4A,D). Similar results were observed regarding eosinophil infiltration (Figure 4B). Thymic stromal lymphopoietin (TSLP) is a cytokine that promotes the homeostasis of CD4+ T cells by acting directly on naive memory CD4+ T cells. This promotes proliferation in response to antigens, thereby playing an important role during the development of inflammatory or allergic reactions [20]. The important role of TSLP in AD aggravation has also been well-described [21]. IHC staining showed that expression levels of TSLP were altered by DNCB application, and this was greatly enhanced when ABX treatment depleted the gut microbiome. However, GJ treatment decreased the TSLP-positive area in the skin, suggesting its beneficial effect on AD (Figure 4A,E). The expression of Ki-67, a proliferative marker which is expressed in the nuclei of dividing cells, was also measured by IHC staining. Consistent with the increased thickness of the skin, Ki-67 was increased in DNCB-treated mice. GJ alleviated the skin thickening, and thus the expression of this proliferation marker was highly suppressed by GJ (Figure 4A,F).

Primed T cells release various cytokines which are programmed to stimulate Th1 cells, Th2 cells, Th17 cells and follicular helper T cells (T_FH_). Among the participating cytokines during this process, IL-12, which plays a pathological role in the development of auto-inflammatory reactions, is derived from dendritic cells (DCs). The activation of Th1 cells produces TNF-α and interferon (IFN)-γ, which mediate a strong inflammatory response. In addition, IL-17 induces the production of several chemokines, and thus amplifies the immune response by promoting T cell invasion and consequent IL-6 release [22]. Increased levels of TNF-α, IL-17 and IL-22 are frequently observed in chronic inflammatory skin diseases, including psoriasis, and IL-17 and IL-22 in particular are reported to have a synergistic relationship during inflammatory responses [23,24]. The gene transcripts of IL-6, IL-12, IL-17A, IL-17F, and IL-22 were measured in the skin of BALB/c mice. From this analysis, it was clear that DNCB upregulated the mRNA level of these genes whether the gut microbiome was depleted or not. However, GJ treatment alleviated this inflammatory response by reducing the mRNA levels of IL-6, IL-12, IL-17A, IL-17F, and IL-22 (Figure 4G–K).

### 3.5. Gardenia jasminoides Extract Reduces Fecal Hemorrhage and Restores Elongated Intestines and Shortened Intestinal Villi to Normal in DNCB-Applied ABX-Induced Mice

**Figure 5:**
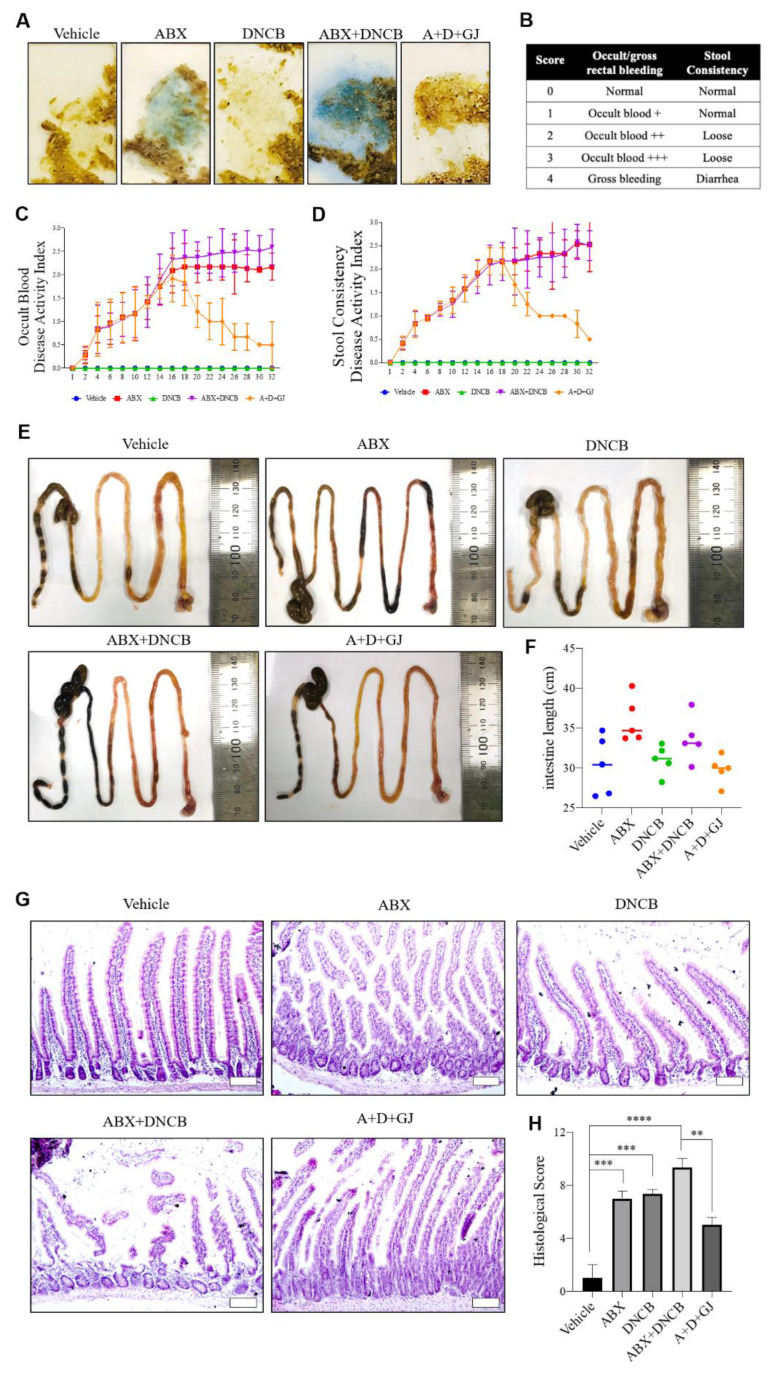
Effect of GJ on pathological changes to the intestine in ABX-induced AD mice. (**A**) Rectal bleeding was measured with a Hemoccult Sensa^®^ kit. Blue coloration indicates rectal hemorrhage. (**B**) Score table proposed based on manufacturer’s guidelines. (**C**) Occult blood DAI and (**D**) stool consistency DAI were evaluated. (**E**,**F**) The length of the intestine of each group was measured. (**G**) H&E staining (magnification ×100) was performed to evaluate intestinal villi length and (**H**) histological scoring of intestinal injury was evaluated. Vehicle: normal control group; ABX: antibiotic cocktail-administered group; DNCB: DNCB-applied group; ABX+DNCB: antibiotic cocktail-administered and DNCB-applied group; A+D+GJ, antibiotic cocktail-administered and DNCB-applied group fed with GJ. ** p < 0.01, *** p < 0.005 and **** p < 0.001.

Fecal occult blood (FOB) is used to quickly screen for colorectal cancer, which cannot be visually confirmed [25]. The evaluation of FOB can also be performed to determine the variance in fecal microbiology and confirm the presence of certain intestinal microflora [26,27]. The Hemoccult SENSA kit was used to investigate the microflora changes in mice. The disease activity index (DAI) was quantified from 0 to 3 points; the higher the score, the more severe the inflammatory responses in the intestine, based on the manufacturer’s instructions [28]. Since the ABX mice continued to consume antibiotics, intestinal inflammation was exacerbated by the depletion of the gut microbiome, resulting in stool hemorrhage. While DNCB application did not affect the DAI, 2 weeks of GJ treatment notably improved the stool health profiles, as determined by a time-dependent gradual decrease in DAI (Figure 5A–D).

Previous studies have shown that the intestine can extend in the ABX model [29], and when the gut microbiome is depleted and loses its balance, discontinuous mucosal epithelium, damaged mucosa, and immune cell infiltration increases [30,31]. After 4 weeks of ingestion of the ABX, the average intestinal length in these mice was increased by 1.3-fold compared to that of control mice, and the intestinal length was also increased in ABX mice with DNCB-derived AD. However, when GJ was administered for 2 weeks, the increase in intestinal length was suppressed, showing a similar length to that of control mice (Figure 5E,F). Furthermore, when observed after H&E staining, the mucous membrane and ileum villi in both DNCB-treated and non-treated ABX mice were noticeably damaged. In the DNCB-induced AD model, the intestinal mucosa showed some damage, but it was confirmed that the number of activated immune cells was increased compared to the control group. The ABX-induced damages were able to be recovered in the intestine of mice fed with GJ, suggesting its protective effect on the intestine (Figure 5G,H).

### 3.6. Gardenia jasminoides Extract Restores Tight Junctions in the Intestine of DNCB-Applied ABX Mice

**Figure 6:**
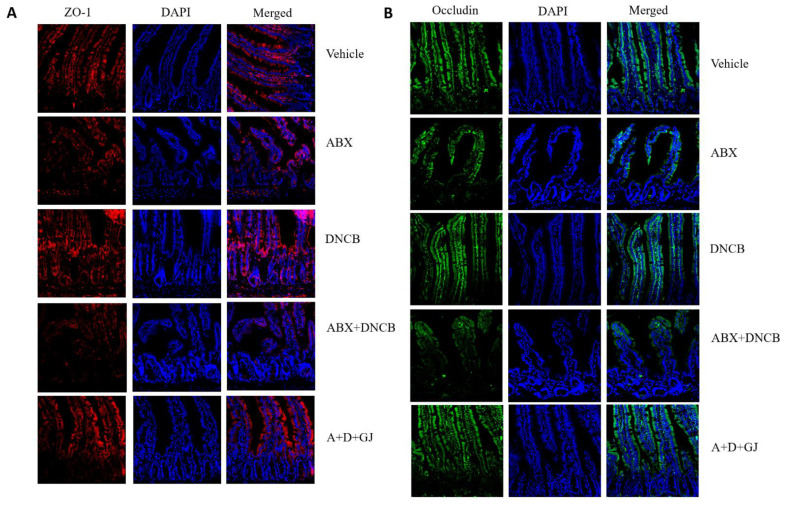
Effect of GJ on intestinal tight junctions in the intestine of ABX-induced AD mice. IF staining (magnification ×200) of (**A**) ZO-1 and (**B**) occludin was performed in the intestinal tissue of mice. Vehicle: normal control group; ABX: antibiotic cocktail-administered group; DNCB: DNCB-applied group; ABX+DNCB: antibiotic cocktail-administered and DNCB-applied group; A+D+GJ: antibiotic cocktail-administered and DNCB-applied group fed with GJ.

The intestine consists of an epithelial monolayer, of which barrier homeostasis is closely related to intestinal health [32]. Under pathological circumstances such as injury, exposure to toxins/inflammatory cytokines, or autoimmune diseases, tight junction proteins are dysregulated, which affects the overall health and function of the intestine [33]. In addition, the intestinal microbiome is also implicated in the control of the integrity of the intestinal barrier [34], including zonula occludens-1 (ZO-1) and occludin [35]. As shown in Figure 6A,B, our results also demonstrated a pathological collapse of tight junctions and subsequent decrease in the expression of ZO-1 and occludin upon the administration of ABX. However, GJ treatment successfully restored these tight junction proteins, implying recovery of the intestinal barrier.

### 3.7. Gardenia jasminoides Extract Suppresses the Expression of Th17-Related Markers in the Intestine of DNCB-Applied ABX-Induced Mice

**Figure 7:**
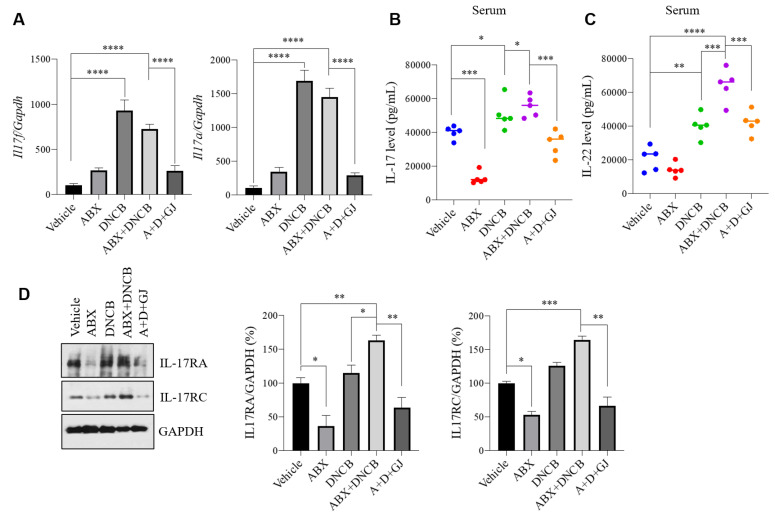
Effect of GJ on Th17-related markers in the intestine of ABX-induced AD mice. mRNA levels of (**A**) *Il17f* and *Il17a* in intestinal tissue of mice were measured by RT-PCR. Serum levels of (**B**) IL-17 and (**C**) IL-22 were measured with ELISA kits. (**D**) Protein levels of IL-17RA and IL-17RC were measured by Western blot. GAPDH was used as an internal control. Vehicle: normal control group; ABX: antibiotic cocktail-administered group; DNCB: DNCB-applied group; ABX+DNCB: antibiotic cocktail-administered and DNCB-applied group; A+D+GJ, antibiotic cocktail-administered and DNCB-applied group fed with GJ. * p < 0.05, ** p < 0.01, *** p < 0.005 and **** p < 0.001.

Th17 cells are important mediators of various immune-related disorders, and among the members of the IL-17 family produced by Th17 cells, IL-17A and IL-17F are the most closely related factors, sharing 55% homology. IL-17A mediates host resistance to extracellular bacterial and fungal infections by acting as a powerful inducer of neutrophil growth factors, such as granulocyte colony stimulating factor (G-CSF) and cytokines IL-1β, IL-9, and IL-12p70. On the other hand, IL-17F plays an important role in the responses of neutrophils against inflammation through the regulation of chemokines and cytokines such as IL-1β, IL-9, and IL-12p70, and GM-CSF release in macrophages [36]. Notably, Th17 responses are considered as a candidate mechanism which links the role of the gut microbiome to the pathology of AD [37]. DNCB-induced AD also displays Th17-related immune responses [7]. Thus, to investigate the response Th17 cells, mRNA levels and protein expression levels of the IL-17 family were measured (Figure 7). The mRNA levels of *Il17f* and *Il17a* showed the tendency to increase in microbiome-depleted mice, but were significantly increased in DNCB-applied mice compared with vehicle control, while ABX mice exposed to DNCB also showed a similar increase in the expression of both genes (Figure 7A). Interestingly, when IL-17 serum cytokine levels were measured, ABX administration resulted in highly suppressed IL-17 expression, but the expression of IL-17 increased when mice were co-treated with DNCB. GJ was able to reduce the overexpression of IL-17 (Figure 7B). The serum level of IL-22 showed consistent changes (Figure 7C). Similarly, the protein levels of the IL-17 receptors, IL-17RA and IL-17RC, were suppressed when the intestinal microbiome was depleted, but were increased upon DNCB application. GJ-treated mice showed expression levels of these proteins at a similar level with normal control mice (Figure 7D). In addition, GJ treatment effectively suppressed the increase of IL-17-related markers, suggesting its beneficial effect in AD.

### 3.8. Gardenia jasminoides Extract Recovers Antibiotic Cocktail- and DNCB-Induced Changes in the Microbiome Composition of DNCB-Applied ABX-Induced Mice

**Figure 8:**
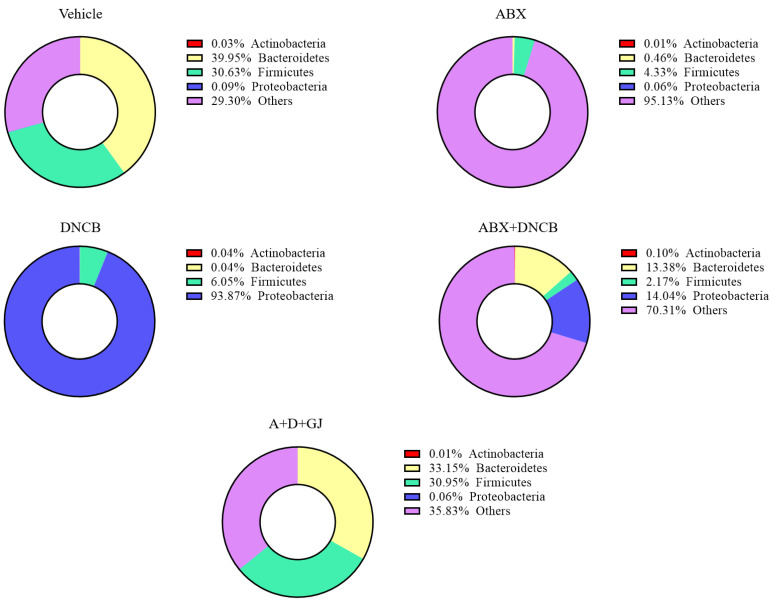
Effect of GJ on gut microbiome composition in ABX-induced AD mice. The shifts in microbiome phylum ratio in each group were investigated by 16s rRNA sequencing. Vehicle: normal control group; ABX: antibiotic cocktail-administered group; DNCB: DNCB-applied group; ABX+DNCB: antibiotic cocktail-administered and DNCB-applied group; A+D+GJ: antibiotic cocktail-administered and DNCB-applied group fed with GJ.

To investigate the microbial environment in the intestine, mouse stool was collected from each group for analysis. In healthy individuals, human and mouse colons contain 10 to 100 trillion (10^14^) microorganisms, with a density of 10^11^–10^12^ cells/mL [38]. When the microbiome is subcategorized according to phylum classification, Firmicutes and Bacteroidetes account for almost 80% of the bacteria present [39]. Accordingly, as shown in Figure 8, the microbial analysis of the stool from control BALB/c mice revealed that Firmicutes and Bacteroidetes were around 70% of the total microbiome. However, this composition of Firmicutes and Bacteroidetes was massively decreased when ABX was administered. The ABX mice showed less than 5% of Firmicutes and Bacteroidetes, accompanied by 95.13% of unclassified microorganisms. The application of DNCB also reduced the ratio of Firmicutes and Bacteroidetes to 6.09%; however, the microbiota of AD mice was composed of nearly 94% Proteobacteria. DNCB-treated ABX mice showed an imbalance in microbiome composition as well. The stool analysis of these mice revealed 0.10% Actinobacteria, 13.38% Bacteroides, 2.17% Firmicutes, 14.04% Proteobacteria, and 70.31% unclassified microorganisms. However, GJ dramatically restored the balance of the gut microbiota, restoring treated mice to 64.10% Firmicutes and Bacteroidetes composition, which is close to the 70.58% found for control mice.

**Figure 9:**
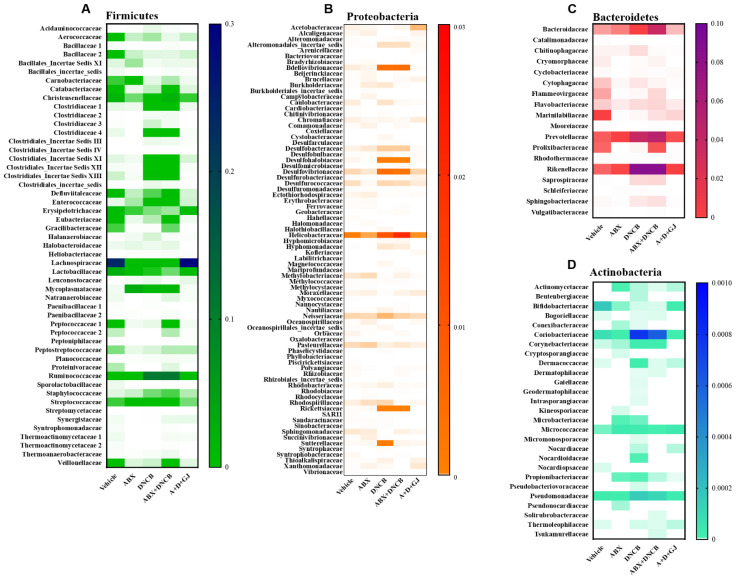
Effect of GJ on Firmicutes, Proteobacteria, Bacteroidetes and Actinobacteria microbiome changes in ABX-induced AD mice. Heatmaps illustrate the composition of the members of the (**A**) Firmicutes, (**B**) Proteobacteria, (**C**) Bacteroidetes and (**D**) Actinobacteria phyla. Each microbiome was characterized with 16S rRNA gene sequencing from stool samples pooled from each group. Vehicle: normal control group; ABX: antibiotic cocktail-administered group; DNCB: DNCB-applied group; ABX+DNCB: antibiotic cocktail-administered and DNCB-applied group; A+D+GJ: antibiotic cocktail-administered and DNCB-applied group fed with GJ.

Next, we analyzed the changes in each subtype of phylum present in the microbiomes of mice. Firmicutes, characterized by Gram-positive cell wall structures [40], are one of the two most abundant types of bacteria that form the gut microbiome, together with Bacteroides. Firmicutes and Bacteroides normally make up over 70% of the total microbiome in a healthy gut [41]. *Bacilli* and *Clostridia* are the two major Firmicutes members in a healthy gut [42]. In the gut of AD patients, the composition or abundance of Firmicutes changes: *Clostridiaceae*, *Ruminococcaceae*, *Staphylococcaceae*, and *Streptococcaceae* increase, and *Erysipelotrichaceae*, *Lachnospiraceae*, *Lactobacillaceae*, and *Thermoactinomycetaceae* decrease [43]. The fold change of Firmicutes in each group was investigated. As shown in Figure 9A, while most members of the Firmicutes family decreased in ABX mice, bacterial species that are known to be increased in inflammatory diseases, such as *Christensenellaceae*, *Clostridiaceae*, *Clostridiales Incertae Sedis*, *Enterococcaceae*, *Ruminococcaceae*, *Staphylococcaceae*, and *Streptococcaceae* were highly increased following DNCB application. On the other hand, *Erysipelotrichaceae*, *Lachnospiraceae*, and *Lactobacillaceae*, which are all considered as beneficial microorganisms, were suppressed in DNCB-induced AD mice regardless of ABX treatment. GJ clearly alleviated this pathologic change in the gut microbiome, restoring the Firmicutes composition to that of vehicle-treated control mice.

The Bacteroidetes phylum is characterized as Gram-negative, non-spore-forming, anaerobic or aerobic and rod-shaped bacteria. They are widely distributed in the environment and are especially abundant in the skin and intestines of animals [44]. In the Bacteroidetes phylum, members related to inflammation include *Bacteroidaceae*, *Porphyromonadaceae*, *Prevotellaceae*, and *Rickenellaceae* [41,45,46]. Our data showed that most Bacteroidetes members were decreased in ABX mice. In DNCB-induced AD mice with or without antibiotic treatment, *Bacteroidaceae*, *Prevotellaceae*, and *Rickenellaceae* were increased, and *Porphyromonadaceae* was decreased. After treatment with GJ for 2 weeks, mice showed that the fold change of these Bacteroidetes was restored to similar levels as seen in the vehicle control mice (Figure 9B).

Proteobacteria is a major phylum that consists of Gram-negative bacteria, including a wide variety of pathogenic genera. The abundance of Proteobacteria including *Bdellovibrionaceae*, *Desulfobacteraceae*, *Desulfohalobiaceae*, *Desulfovibrionaceae*, *Enterobacteriaceae*, *Helicobacteraceae*, *Rickettsiaceae*, and *Sutterellaceae* is reported to increase in inflammation [46,47,48,49]. As shown in Figure 9C, DNCB application altered the levels of these Proteobacteria; however, in GJ-treated mice, the composition of *Bdellovibrionaceae*, *Desulfobacteraceae*, *Desulfohalobiaceae*, *Desulfovibrionaceae*, *Enterobacteriaceae*, *Helicobacteraceae*, *Rickettsiaceae*, and *Sutterellaceae* was suppressed back to their normal levels.

Actinobacteria is a group of Gram-positive bacteria which accounts for a rather small proportion of the intestinal microbial composition; however, this phylum plays a central role in microbial homeostasis. Almost all Actinobacteria are involved in microbial homeostasis, yet some are pathogens that induce inflammation while some are beneficial bacteria [50,51,52]. Among the various members of the Actinobacteria phylum, *Corynebacteriaceae* and *Propionibacteriaceae* are the most important cocci associated with the skin microbiota profile, and *Coriobacteriaceae*, *Nocardioidaceae*, and *Pseudomonadaecae* are also reported to be important inflammation-related microorganisms [52,53]. As shown in Figure 9D, DNCB-applied AD mice showed increased *Coriobacteriaceae*, *Corynebacteriaceae*, *Propionibacteriaceae*, *Nocardioidaceae*, and *Pseudomonadaecae*, and decreased *Bifidobacteriaceae*, suggesting that AD induced an imbalance in gut microbiome homeostasis. GJ treatment, on the other hand, reversed this imbalance by suppressing the increase in *Coriobacteriaceae*, *Corynebacteriaceae* and *Propionibacteriaceae*, *Nocardioidaceae*, and *Pseudomonadaecae*, and restoring *Bifidobacteriaceae*.

## 4. Discussion

The balance of the gut microbial community is an important component that plays a large role in health and disease [54,55]. Disintegration of the microflora can cause various diseases, including metabolic diseases, inflammation, and cancer; therefore, the homeostasis of the microbial community is very important in tissues wherever microorganisms exist [56,57,58,59]. In particular, more research is required on the exact role of microbiomes in inflammation, as it has the possibility to lead to the development of other diseases by triggering an autoimmune response. The role of the intestinal microflora has been studied in various models of inflammatory and autoimmune diseases [60,61,62,63]. The innate immune response by the microflora is relatively well-described due to intense research on the functional properties of the microorganisms involved in mucosal immunity and distal inflammation [4]. The intestinal microbiome contributes to the development of AD, as it has been shown to be associated with onset and severity of AD [5]; a low diversity in the intestinal microbiome is clearly linked to the epidemiology [64] and severity [65] of AD. The decreased diversity or abundance of Bacteroidetes has been observed in infants with atopic eczema [64,66]. Studies which report the importance of the intestinal microbiome in an AD mouse models reflect these clinical findings. Fecal microbiomes are correlated with pro- and anti-inflammatory cytokines, even before the induction of skin inflammation, and the composition of the intestinal microbiome was found to be altered upon oxazolone-induced AD [67]. Moreover, Debes et al. recently showed that intestinal microbiome transplants to germ-free mice reflected the AD-responding phenotype from donor mice [68]. In another study, ovalbumin-induced AD was aggravated in mice with intestinal microbiome dysbiosis established by the oral administration of antibiotics [69]. These studies imply the important role of the intestinal microbiome in allergic responses such as AD, and thus allow us to estimate the impact of gut microbiome composition on health and also provide a promising potential therapeutic option for such diseases [70]. Almost 80% of the microflora in a healthy intestine is composed of Firmicutes and Bacteroidetes, and when an imbalance occurs due to inflammation, the composition of the intestinal microflora changes. The results from this study fall in line with the literature. Through the administration of an ABX, the intestinal microbiome balance was damaged, showing less than 5% Firmicutes and Bacteroidetes composition compared to 70.58% in healthy control mice. In DNCB-induced AD mice, Firmicutes and Bacteroidetes made up around 6% of the total microflora, accompanied with 94% Proteobacteria, and in ABX mice with AD, 15.55% Firmicutes and Bacteroidetes were found, with 14.04% Proteobacteria. The massive changes in these groups imply the importance of the gut microbiome in the pathology of AD.

AD is a complex multifactorial disease that accompanies barrier dysfunction, changes in cell-mediated immune responses, and IgE-mediated hypersensitivity [17]. The immune response occurs due to the imbalance of Th2, Th1, and Th17 cells. The primary function of Th1 and Th2 cells is in host defense, whereas Th17 cells are involved in the inhibition of inflammatory mediators [71]. Th17 cells allow B cells to generate antibodies, kill macrophages and microbes, and activate other immune cells at the site of infection or inflammation [72]. In the pathogenesis of AD, the balance between Th1 cell cytokines (IFN-γ, IL-12, and TNF-α), Th2 cytokines (IL-4, IL-6, IL-10, IL-13, and IgE), and Th17 cytokines (IL-17 and IL-22) is known to regulate inflammatory responses in the skin. In particular, it has been reported that IL-17 weakens the junction barrier [73,74,75]. It has also been reported that GJ improves AD-like lesions by increasing the expression of skin barrier proteins and inhibiting Th2 inflammatory responses [76], and its main component (crocin) also exerts anti-inflammatory effects by suppressing IgE levels, thereby reducing the infiltration of immune cells, suppressing cytokine release and the activation of NF-κB to improve AD symptoms [12]. In addition, Treg cells play an important role in the pathogenesis of AD [77]. CD4+ Treg cells modulate excessive immune responses by secreting anti-inflammatory cytokines or by controlling other immune cells, such as dendritic cells or Th cells [78]. Treg cells are also involved in experimental AD models, as implicated in several studies [79,80,81]. Moreover, the intestinal microbiome is also related to Treg cells as its depletion interrupts colonic Treg cell development [82]. This reflects the close link between Treg and Th17 cells [83], which plays a crucial role not only in immune responses, but also in the integrity of intestinal health. It was reported by Liao et al. that geniposide, a component of GJ, increased splenic Treg cells and thus possessed a beneficial immunoregulatory effect [84]. In line with this, in the DNCB-induced AD mouse model established in this study, GJ treatment alleviated AD-like symptoms such as skin condition, thickness, DOA, and epidermal thickness. IgE, TNF-α, IL-12, and IL-6 were also increased in the serum of DNCB-derived AD mice.

IL-17, a family of six cytokine members secreted mainly by Th17 cells, has been demonstrated to be a predominantly pro-inflammatory cytokine [85]. However, its function may be multifaceted in certain situations. One of the most important functions of IL-17 specifically regarding intestinal health is its role during the protective immune response at mucosal sites [86]. IL-17 is also known to regulate the microbiome composition, and vice versa. For instance, it was shown by Hill et al. that depletion of the intestinal microbiome led to suppressed IL-17A expression [87], which was replicated in our study. On the other hand, disruption of IL-17R signaling in the intestine resulted in dysbiosis of segmented filamentous bacteria, which subsequently weakened the immune function of the intestine [88]. Our results may have reflected this phenomenon. ABX administration resulted in a massive reduction of serum IL-17, but GJ administration increased serum IL-17 levels up to the normal levels, and therefore restored the microbiome composition. On the other hand, we observed that IL-17A and IL-17F genes were increased in the gut tissue of ABX mice. The lack of IL-17 may have accelerated the transcription of *Il17* mRNA via positive feedback. GJ treatment restored serum IL-17 back to normal levels, which in turn would have negatively affected mRNA levels [89]. Similar to the results of the cytokine assay, the expression of the IL-17 receptors IL-17RA and IL-17RC were induced by DNCB, regardless of the administration of ABX. This inflammatory response was reversed by GJ treatment, suggesting its anti-inflammatory effect and its ability to regulate the balance of the microbiome within the intestine. Another cytokine, IL-22, is highly expressed by Th17 cells [90] and is considered important in intestinal health. IL-22 has been reported to control genes that are involved in tissue protection [91]. Unlike IL-17, the level of IL-22 is maintained in the intestine of germ-free mice; however, its absence leads to exacerbated inflammation [92,93], suggesting the protective role of this cytokine. Yet, IL-22 is considered as a pro-inflammatory cytokine and is induced in the inflamed colon [94]. We observed that GJ treatment suppressed the DNCB-induced overexpression of IL-22 in the serum.

The epithelial layer and the intestinal microflora surrounding the intestinal lumen are responsible for digestion and the absorption of nutrients. In addition to the digestion/absorption process, they also serve as a barrier to maintain the inside and outside environment of the intestine [58]. Studies on mucosal immunology are being actively investigated [95], and have so far revealed some interesting insights. For example, when the mucosal barrier is damaged, pathogens enter the mucosa, which leads to the pathogenesis of AD [96]. Therefore, stabilization of the microorganisms in the intestine is vital as they may interact with microorganisms in the skin to benefit each other [97]. In this study, the total health profile within the intestine was investigated. First, the health status of the intestine was examined using the Hemoccult SENSA test, which suggested an improvement as a result of GJ treatment to a stabilized healthy status. While the balance of the intestinal microbiome was damaged in AD-induced mice, GJ significantly restored the balance back to normal levels. In addition, the decrease of pathogens belonging to Firmicutes, Bacteroidetes, Proteobacteria, and Actinobacteria phyla in the gut was restored to normal levels by GJ treatment, and beneficial organisms such as *Erysipelotrichaceae*, *Lachnospiraceae*, *Lactobacillaceae*, *Porphytomonadaceae*, and *Bifidobacteria* were increased. These results suggest that the beneficial effect of GJ in AD involves its action on the gut microbiome.

There may be several explanations for the effect of GJ on the intestinal microbiome and the subsequent alleviation of AD symptoms, especially when we focus on its components. Geniposide, in combination with chlorogenic acid, has been reported to display protective effects on gut barrier function by increasing the expression of ZO-1 and occludin to ameliorate non-alcoholic fatty liver disease [98]. Crocin, identified in GJ by LC-MS-MS, restored the composition of Bacteroidetes in a mouse model of disrupted lipid metabolism and dysbiosis caused by chronic corticosterone administration [99]. In addition, crocin treatment also recovered the expression of tight junction proteins (ZO-1, occludin, and claudin-1) and microbiome composition (particularly the increase of Bacteroidetes and Proteobacteria), and eventually alleviated depression-like behaviors [100]. Moreover, although involving other phyla, it was also reported that another component of GJ, crocetin, increased the abundance of *Turicibacter*, *Romboutsia* and *Alistipes* in the intestinal microbiome [101]. Rutin supplementation in food led to an increase of Bacteroides proportion in mice [102], which was also shown in an in vitro mixture of human microbiota as well [103]. In addition to the effect on the intestinal microbiome, rutin also alleviated microbial dysbiosis, colonic tissue damage, and disease symptoms in a dextran sodium sulfate-induced colitis model [104]. Based on these previous studies, we expect these components that we have identified through LC-MS-MS are responsible for the beneficial action of GJ on the intestinal microbiome composition. However further investigation is required to reveal the roles of others components, such as jasmigeniposide A, picrocrocinic acid, shanzhiside, gardoside, etc., and even the possible interactions between these components.

In conclusion, this study reports that the beneficial effect of GJ on AD involves its ability to restore balance to the intestinal microflora profile. GJ administration improved the overall symptoms of AD, including skin and epidermal thickness and the expression of key cytokines; most importantly, GJ restored the microflora composition in the intestine to a normal status. The composition of the microflora can be affected by various factors, including the environment and diet [61]. However, it is possible to stabilize the balance of the intestinal microflora with probiotics composed of beneficial bacteria and prebiotics that promote the growth of specific bacteria [4,63,70,95]. GJ was shown to have a corresponding effect. Therefore, we suggest GJ as a potential therapeutic agent for AD, as it exerts its effect not only by affecting the inflammatory responses of immune cells, but also by improving the intestinal microbiome profile.

## Figures and Tables

**Table 1:** LC-MS-MS conditions.

Parameter	Condition		
Flow Rate	1.0 mL/min		
Injection Volume	10 µL		
Column	YMC Pack-Pro C18		
Column Temp.	30 °C		
Heater Temp.	250 °C		
Sheath Gas Flow Rate	35 arb (N_2_)		
Spray Voltage	5 kV		
Capillary Temp.	275 °C		
Gradient Conditions	Time (min)	Water (0.1% formic acid)	Acetonitrile (0.1% formic acid)
	0	95	5
	10	95	5
	50	30	70
	55	10	90
	65	95	5

**Table 2:** Mouse PCR primer sequences for RT-PCR.

Primer Name	Sequence (5′->3′)	
*Il6*	Forward	GATGCTACCAAACTGGATATAATC
	Reverse	GGTCCTTAGCCACTCCTTCTGTG
*Il12*	Forward	ATGGCCATGTGGGAGCTGGAG
	Reverse	TTTGGTGCTTCACACTTCAGG
*Il13*	Forward	CGGCAGCATGGTATGGAGTG
	Reverse	ATTGCAATTGGAGATGTTGGTCAG
*Il17a*	Forward	ATCAGGACGCGCAAACATGA
	Reverse	TCAAAGCTCAGCGTGTCCAA
*Il17f*	Forward	TGCTACTGTTGATGTTGGGAC
	Reverse	TTCAACCAAAACCAGGGCATT
*Il22*	Forward	TTGAGGTGTCCAACTTCCAGCA
	Reverse	AGCCGGACATCTGTGTTGTTA
*Gapdh*	Forward	GAGGGGCCATCCACAGTCTTC
	Reverse	CATCACCATCTTCCAGGAGCG

**Table 3:** Histological scoring system of intestinal injury.

Grade	Severity of Inflammation	Extent of Inflammation	Crypt Damage
4	–	–	Crypt and surface epithelium lost
3	Severe	Transmural	Crypts lost, surface and epithelium present
2	Moderate	Mucosa and submucosa	2/3 damages
1	Mild	Mucosa	1/3 damages
0	None	None	None

**Table 4:** Proposed structures by LC-MS-MS analysis.

No.	Retention Time (min)	Positive/Negative Mode	Molecular Weight	Proposed Structure
1	2.88	[M-H]+	387.18	geniposide
2	11.06	[M-H]+	723.43	jasmigeniposide A
3	16.50	[M-H]+	329.17	crocetin
4	18.44	[M-H]+	347.09	picrocrocinic acid
5	18.47	[M-H]+	391.26	shanzhiside
6	23.76	[M-H]+	373.26	gardoside
7	24.47	[M-H]+	609.26	rutin
8	25.58	[M-H]+	561.35	caffeoyl sinapoyl quinic acid
9	27.40	[M-H]+	725.35	6”-*O*-*trans*-feruloyl genipin gentiobioside
10	28.33	[M-H]+	659.35	3,4-dicaffeoyl-5-(3-hydroxy-3-methylglutaroyl) quinic acid
11	31.18	[M-H]+	725.35	6”-*O*-*trans*-cinnamoyl genipin gentiobioside
12	32.70	[M-H]+	975.52	crocin

## Data Availability

The data presented in this study are available on request from the corresponding author.
